# Advancements in artificial intelligence for the localization of premature ventricular contraction origins

**DOI:** 10.3389/fcvm.2026.1864886

**Published:** 2026-06-29

**Authors:** Changyu Wang, Zhiqiang Pei, Xingxing Cai

**Affiliations:** 1 School of Health Sciences and Engineering, University of Shanghai for Science and Technology, Shanghai, China; 2 Oriental Pan-Vascular Devices Innovation, University of Shanghai for Science and Technology, Shanghai, China; 3 Xinhua Hospital Affiliated to Shanghai Jiao Tong University School of Medicine, Shanghai, China

**Keywords:** algorithm models, artificial intelligence, electrocardiogram, origin localization, premature ventricular contractions

## Abstract

Premature ventricular contraction (PVC) is one of the most common types of arrhythmias, and accurately locating the origin sites of PVC is the key to establishing catheter ablation strategies. However, traditional manual analysis methods that rely on electrocardiogram (ECG) features are highly subjective and inefficient, making it difficult to meet the demands of clinical precision treatment. The emergence of artificial intelligence (AI) has provided new opportunities to address these limitations through automated analysis of complex ECG data. This review traces the evolution of ECG-based PVC localization criteria, summarizes recent advances in AI algorithm models for identifying PVC origins. Recent studies have shown that AI-based approaches can improve the efficiency and accuracy of PVC origin identification by extracting high-dimensional features from ECG data and enabling automated classification of different origin sites. This review discusses the prospects and challenges of AI application in the precise diagnosis of PVC.

## 1 Introduction

Premature ventricular contraction (PVC) is one of the most common types of arrhythmias in clinical practice, with a prevalence of approximately 1%–4% in the general population (1). While PVC is common and typically has a favorable prognosis in patients without structural heart disease (SHD), the prognosis of PVC is considerably poorer in patients with coexisting organic heart disease (2). Frequent and high-burden PVCs may lead to ventricular dilation and impaired cardiac function, and may even trigger malignant arrhythmias such as ventricular tachycardia or ventricular fibrillation. Therefore, finding the precise origin sites of PVC is crucial for performing effective clinical management and reducing the risk of cardiac sudden death.

Currently, pharmacological treatment and interventional therapy are the main options for alleviating PVC-related symptoms. However, pharmacological treatment is often unable to completely eliminate PVCs, and long-term use may be associated with significant adverse drug events and might even increase the risk of malignant arrhythmias. Hence, catheter ablation is an effective curative treatment for patients with a high PVC burden (≥15%) or refractory to medical therapy (3). Moreover, the effective catheter ablation process depends on accurate PVC origin localization, which not only optimizes the ablation strategy but also reduces complications and shortens the duration of procedure.

In recent years, the application of AI in medical practice has been growing rapidly. A key characteristic of AI is its ability to learn from data and adapt to new information without explicit programming for each task. Machine learning (ML) represents a major approach within AI, enabling algorithms to identify patterns and relationships in datasets and to construct predictive models. Deep learning (DL), a specialized branch of ML, employs multi-layered neural networks to automatically extract hierarchical features from raw data, capturing complex dependencies that are difficult to define manually. As a major branch of computer science, AI applies these methods to perform tasks involving pattern recognition and data analysis (4). As a result, AI has created new opportunities in the field of clinical medicine and facilitated advances in cardiac electrophysiology (5).

The electrocardiogram (ECG) is the crucial tool for analyzing the localization of PVC origin. However, traditional ECG-based analysis methods rely on physician experience and manual feature extraction, which are hampered by high subjectivity and low efficiency. AI methods, particularly those based on deep learning (DL) approaches, provide a systematic framework to automatically decode the complex information hidden within ECG data and extract hierarchical features. These approaches improve the accuracy and efficiency of PVC origin localization, offering a feasible approach for the analysis of ECG big data and enhancing the accuracy of clinical PVC identification.

In this article, we reviewed research progress of AI application for localizing the origin of PVC based on 12-lead ECG data, and discussed the technical advantages, application prospects and existing challenges. The aim of this review is to provide a theoretical foundation and practical guidance for clinical translation of AI-based PVC origin localization.

## 2 Localization of PVCs originating from the outflow tract

Accurate localization of the origin of PVC in the outflow tract is critical to the success of catheter ablation. Traditional ECG-based criteria rely on manually derived parameters, such as the transition zone index, the V2S/V3R ratio, and the S-R amplitude difference. While these criteria are useful, they are subjective, time-consuming, and have limited coverage of anatomical variations. In comparison, AI methods, particularly machine learning (ML), including its specialized branch DL, can automatically extract discriminative features from large-scale ECG datasets, significantly improving accuracy and reproducibility. AI models have demonstrated superior performance in distinguishing between left and right ventricular outflow tract origins and have been extended to identify subtle patterns such as PVCs originating from the left ventricular summit. Below, we detail the evolution of traditional ECG criteria and recent advances in AI-based outflow tract PVC localization.

### 2.1 Traditional ECG localization

The outflow tracts are the most common sites of origin for PVCs, with the right ventricular outflow tract (RVOT) accounting for 60% to 80% of cases (6). Because the left ventricular outflow tract (LVOT) and the RVOT are anatomically adjacent, PVCs originating from either tract exhibit similar patterns on surface ECGs and are difficult to distinguish based solely on bundle branch block patterns (7). In recent years, researchers have developed a series of manual algorithms based on 12-lead ECG features to distinguish between RVOT and LVOT origins.

In 2002, Ouyang et al. proposed that the R-wave duration and R/S amplitude index in leads V1 or V2 could aid in the identification of outflow tract PVCs: PVCs originating from the LVOT typically show an anterior lead transition in leads V1 or V2, whereas those originating from the RVOT typically show an anterior lead transition in lead V4 or beyond. However, this algorithm did not account for the effects of cardiac transposition, which may reduce its predictive performance (3). Subsequently, Yoshida et al. introduced the transition zone index (TZ index), which is defined as the difference between the precordial transition zone during PVCs and that during sinus rhythm. The precordial transition zone refers to the lead at which the ratio of R-wave amplitude to S-wave amplitude in the precordial leads falls between 0.9 and 1.1. A TZ index < 0 suggests an LVOT origin, while a TZ index ≥ 0 suggests an RVOT origin (8). Betensky et al. proposed the V2 transition index (defined as the ratio of the percentage of R-wave amplitude in PVCs to that in sinus beats in lead V2, calculated as {[PVC R/(R + S)]}/{[sinus R/(R + S)]}, with a value ≥0.60 achieving 91% accuracy in predicting LVOT (9). As research progressed, Yoshida et al. developed the V2S/V3R index (≤1.5 suggests LVOT, with a sensitivity of 89% and specificity of 94%) based on the anatomical characteristics (10). Kaypakli et al. distinguished the origin by analyzing the S-R amplitude difference in the V1-V2 leads, defining as the sum of S-wave amplitudes in leads V1 and V2 minus the sum of R-wave amplitudes during PVCs, a value >1.625 mV suggests RVOT (11). Xia et al. further found that an amplitude of ≥0.52 mV during the first 40 ms of the QRS complex in lead V2 can serve as a diagnostic criterion for LVOT origin (12). To overcome the effects of electrode placement and cardiac rotation, Di et al. proposed the V1-V3 transition zone index. The index is calculated as follows: the S-wave amplitudes of PVCs in leads V1 and V2 are summed, each divided by the S-wave amplitude of the same lead during sinus rhythm. Then the sum of the R-wave amplitudes of PVCs in leads V1, V2, and V3 is subtracted from the R-wave amplitude of the sinus beat in the same lead. Finally, the sum of these results is subtracted. A value >−1.60 suggests an RVOT origin (13).

The evolution of these algorithms reflects a transition from single indices toward composite parameters and from empirical judgment toward anatomically correlated localization. However, they still rely on manual feature selection, which is not only limited in scope but also susceptible to subjective factors. The key traditional ECG criteria for distinguishing LVOT from RVOT origins are summarized in Table 1. For each criterion, the definition, suggested cutoff, reported sensitivity, and specificity are listed.

**Table 1:** Traditional ECG criteria for differentiating left vs. right ventricular outflow tract PVC origins.

Method	Expression	Author	Distinguishing point	Specificity	Sensitivity
R-wave duration & R/S amplitude index	R-wave duration and R/S ratio in leads V1 or V2	Ouyang (2002)	LVOT: R-wave duration ≥50% and R/S amplitude index ≥30%	–	–
Transition zone (TZ)	TZ_PVC_-TZ_SR_	Yoshida (2011)	LVOT: <0	82%	88%
V2 transition index	{[R_PVC_/(R + S)]}/{[R_SR_/(R + S)]}	Betensky (2011)	LVOT: ≥0.6	100%	95%
V2S/V3R index	S-wave amplitude in V2/R-wave amplitude in V3	Yoshida (2014)	RVOT: >1.5	94%	89%
V1 -V2 S-R amplitude difference	(SV1 + SV2) − (RV1 + RV2 + RV3) during PVC	Kaypakli (2018)	RVOT: >1.625	86%	95%
V1-V3 transition index	[(S_PVC_/S_SR_)V1 + (S_PVC_/S_SR_)V2] − [(R_PVC_/R_SR_)V1 + (R_PVC_/R_SR_)V2 + (R_PVC_/R_SR_)V3]	Di (2019)	RVOT: >−1.6	86%	93%
V2QRSi40	Amplitude of QRS complex within the initial 40 ms in lead V2	Xia (2020)	LVOT: >0.52 mV	93%	84%

PVC, premature ventricular contraction; SR, sinus rhythm; TZ, transition zone; −, not reported.

### 2.2 AI-based localization

To address the limitations of traditional ECG-based methods, AI-based approaches have been developed to improve the accuracy and efficiency of PVC localization. The application of AI in localizing outflow tract PVC has evolved across both traditional ML models and DL architectures. As a subfield of ML, DL utilizes multilayer neural networks to automatically learn complex ECG features from raw signals. Unlike traditional methods, which are limited by reliance on manually engineered features, DL models use convolutional neural networks (CNNs) to process 12-lead electrocardiograms as spatiotemporal matrices. This approach facilitates the automatic extraction of hierarchical features in both the temporal and lead domains, capturing complex inter-lead relationships that are often overlooked in human interpretation. Consequently, these models have demonstrated superior performance in identifying subtle ECG phenotypes associated with specific anatomical origins.

AI-based localization of outflow tracts PVCs was characterized by high-dimensional feature engineering. In a representative study by Zheng et al. (14), a proprietary algorithm was employed to extract over 1.6 million features from 12-lead ECG data (14). High accuracy in differentiating left from right ventricular outflow tract origins was achieved within the single center in-house development cohort. The study was limited by the absence of external validation. Consequently, the model's generalizability to heterogeneous populations or patients with SHD remains unverified. Moreover, the algorithm's black-box nature, combined with the lack of independent validation, is associated with potential limitations for clinical translation due to the risk of overfitting.

Early AI models achieved high predictive accuracy. However, they relied on high-dimensional feature extraction and lacked interpretability, which limited clinician trust and hindered clinical adoption. To address these limitations, subsequent studies shifted focus from predictive performance to enhancing model interpretability. Nakasone et al. (15) integrated Grad-CAM with a CNN architecture to visualize the spatiotemporal regions contributing to the model's decisions (15). Their main contribution was to elucidate the decision-making process, enhancing clinician trust in black-box algorithms. While such retrospective explanations are valuable in scenarios requiring diagnostic justification, they do not mitigate the underlying requirement for large, well-curated datasets. Furthermore, the generalizability of these findings remains constrained by the reliance on a single center cohort.

Recognizing the limitations of early models, more recent studies have emphasized the integration of clinical knowledge to simplify model design while maintaining clinical relevance. Shimojo et al. (16) developed a practical ML algorithm to differentiate left and right outflow tract ventricular arrhythmia origins. In this study, 128 ECG features were measured. A subset of the most informative parameters, including ratios and amplitudes in lead V3 and other relevant leads was selected using a decision tree classifier (16). This model was found to achieve high accuracy in a single center cohort, and because it relies on a modest number of feature measurements rather than a deep network, it is computationally efficient and easier to interpret than many DL models. However, its performance on complex or atypical ECG morphologies remains to be determined. Its accuracy in independent cohorts has not yet been evaluated.

Recently, Kujime et al. (17) developed a DL-based semantic segmentation model for PVC origin localization using 12-lead ECGs (17). In their single center study of 84 patients (265 recordings), the model achieved 93% overall accuracy, with an F1-score of 94.5%. Performance was limited by class imbalance, particularly for rare PVC origins such as the LV summit and papillary muscles. The model may not generalize to patients with SHD. In external validation using a public dataset of 334 patients, the model achieved 91.6% accuracy and an F1-score of 94.3%, while the rate of neutral cases (cases without confident prediction) increased to 28%. The method provides probabilistic temporal maps to assist clinical decision-making and identifies the spatiotemporal regions of the ECG that contribute most to predicted PVC origins. Further validation in diverse patient populations is necessary to confirm the model's generalizability.

Another study constructed the largest and most balanced test dataset and developed a model integrating left/right outflow tract classification with left ventricular summit (LV Summit) identification (18). This model demonstrated improved accuracy in identifying PVCs originating from the summit region, indicating enhanced clinical relevance compared to classifications that identify only RVOT or LVOT. External validation has not yet been performed. To further enhance localization accuracy, some studies have explored the application of high-resolution ECG (19). However, its widespread adoption remains limited by factors related to device accessibility and algorithm maturity.

**Figure 1:**
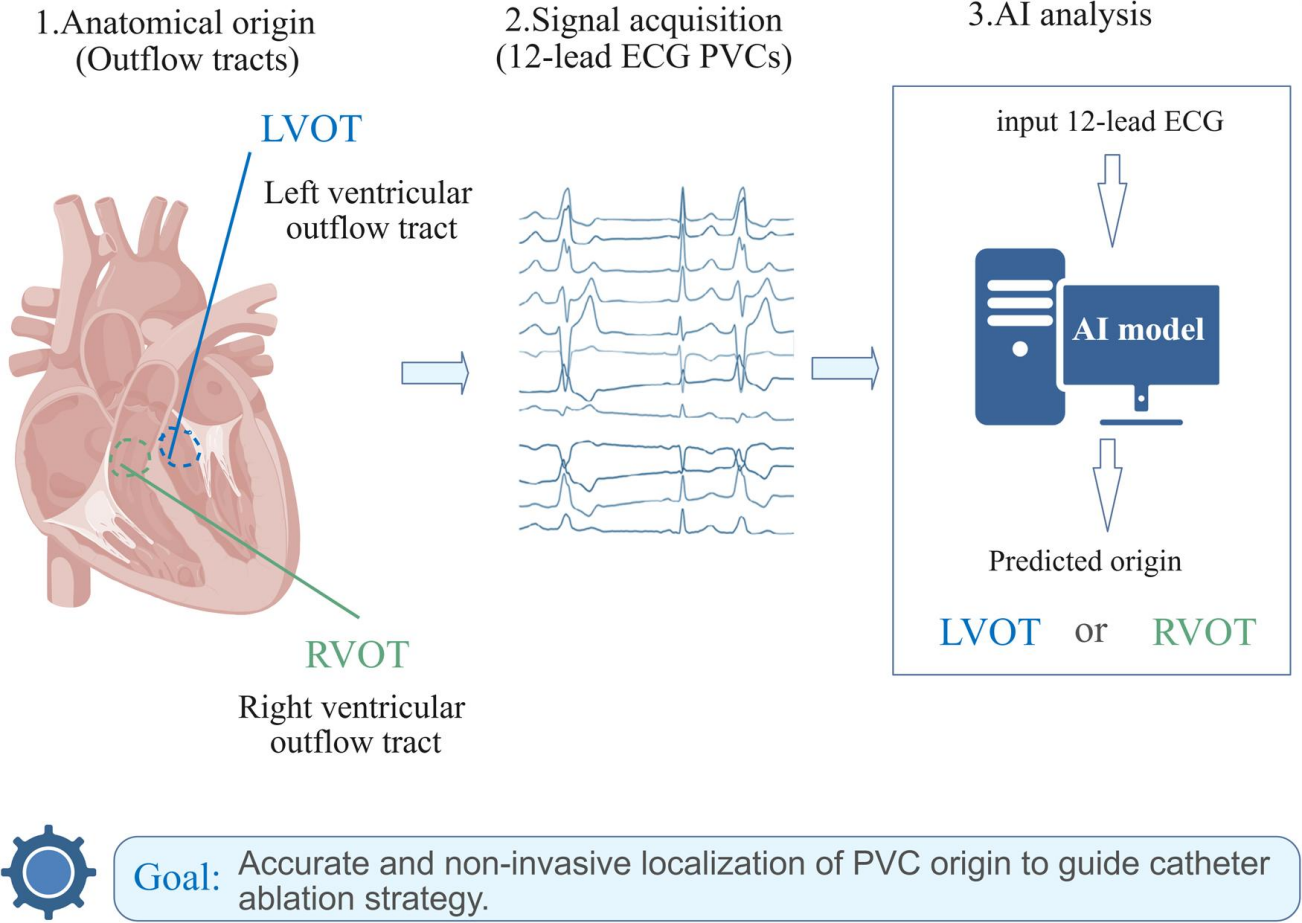
The process of localizing PVC origins from RVOT or LVOT by AI.

In summary, AI has demonstrated high accuracy in predicting PVCs originating from the outflow tract in existing single center studies, but individual anatomical variations, SHD condition, and data imbalance limit the generalizability of these models. Moreover, mature AI localization models for key outflow tract subregions, such as the free wall and septal region, are currently lacking, which hinders precise ablation guidance. Figure 1 illustrates the workflow of PVC origin localization.

## 3 Localization of PVCs originating from non-outflow tract

Localizing non-outflow tract PVCs is more challenging because their ECG morphology often overlaps with that of outflow tract PVCs and exhibits greater interindividual variability. Traditional ECG criteria identify cardiac origins such as the tricuspid annulus, papillary muscles, or the right bundle branch by relying on specific waveform patterns, including QRS notches, R wave amplitude ratios and lead-specific indices. Although these rules are useful, they are derived from limited case series and lack generalizability. In contrast, AI-based approaches, including ML methods such as support vector machines (SVM) and DL methods, can automatically learn high-dimensional ECG features and demonstrate significantly higher accuracy in classifying PVCs with these rare origins. By integrating automated feature extraction and hierarchical classification, AI can more accurately and non-invasively identify challenging non-outflow tract PVC origins, thereby facilitating personalized ablation strategy development. The subsection further compared the traditional ECG estimation with the AI-based analysis in the localization of non-outflow tract PVCs.

### 3.1 Traditional ECG localization

Although most PVCs originate in the RVOT/LVOT, a small number of cases originate in other locations. Recent studies have shown that PVCs originating outside the outflow tracts also exhibit certain characteristic ECG patterns, which can be used to make a preliminary determination of their origin.

PVCs originating from the tricuspid annulus are primarily concentrated in the anterior septal region near the bundle of His. Their ECG characteristics are similar to those of PVCs originating from the RVOT. In most cases, they can be distinguished by the direction of the main QRS complex in lead aVL (20). A study by Jin W et al. (21) showed that PVCs originating from the tricuspid annulus frequently exhibit QRS notches in the inferior leads and leads V4-V6, and the R-wave amplitude in lead I is higher than that during sinus rhythm. In contrast, PVCs originating from the RVOT rarely exhibit QRS notches, and the R-wave amplitude in lead I is lower. Lu et al. (22) further noted that PVCs originating at the junction of the RVOT and the tricuspid annulus exhibit unique ECG characteristics: unidirectional R waves in lead I and the inferior leads, and a significantly lower QRS amplitude in lead aVL compared to those originating from the tricuspid annulus (0.3 ± 0.1 mV vs. 0.8 ± 0.3 mV, *P* < 0.05). In addition, PVCs originating from the right bundle branch show lower R-wave amplitude in the aVF lead and shorter QRS duration in the inferior leads on the ECG, while the R-wave amplitude is higher in leads I, V5, and V6, and the V1 lead typically exhibits a QS pattern (23).

Komatsu et al. (24) proposed that if the ECG meets the following conditions, the R-wave amplitude in lead I is >0.45 mV, the mean R-wave amplitude in inferior leads is <0.91 mV, the ratio of the R-wave amplitudes in leads III and II is <0.67, an S-wave is present in lead III, and lead V1 shows a QS pattern, this strongly suggests a likely origin below the His bundle in the mid-septum of the right ventricle. Conversely, if these characteristics are not met, the origin is more likely to be above the His bundle in the anterior septum of the right ventricle. Li et al. (25) revealed that PVCs originating from the left ventricular papillary muscles showed QRS duration typically >160 ms, whereas QRS duration for those originating from the left anterior fascicle is <130 ms. When the QRS duration is between 130 and 160 ms, further differentiation could be made using the 40 ms excitation rate ratio between the onset and end of the QRS wave (a ratio ≤ 0.85 suggests origin from the left anterior fascicle). Briceno et al. (26) demonstrated that the presence of an Rr wave, an R wave with a notched descending limb, or an RR-type QRS complex in lead V1 suggests a left ventricular papillary muscle origin. Chang et al. (27) proposed that if the ratio of the time from the onset to the peak of the R wave in lead I to the total QRS duration is ≥0.5, this indicates a distal origin in the great veins of the heart.

### 3.2 AI-based localization

Localizing non-outflow tract PVCs is more challenging than localizing outflow tract PVCs, due to their more scattered anatomical origins and higher interindividual variability in ECG morphology. Thus, AI research in this domain faces increased complexity. The field has followed a clear path, from traditional ML (classification and regression) to DL (end-to-end, fine-grained recognition). These two approaches serve different clinical needs. While traditional ML offers a practical means for broad ventricular mapping, DL is better suited for pinpointing specific anatomical substrates.

Early efforts in this domain relied heavily on these traditional ML techniques, utilizing manual feature engineering and regression models to obtain a rough estimate of the PVC origin. Yokokawa et al. (28) pioneered the integration of ML with ventricular segmentation and localization by using a SVM algorithm to classify PVCs into 10 left ventricular segments based on digitized pacing waveforms. With technological advancements, Sapp et al. (29) proposed two innovative methods in 2017, template matching and ensemble-based multiple linear regression, in order to automatically identify the segments and locations of left ventricular excitation. By subdividing the left ventricle into 16 anatomical segments using a discrete localization method, they achieved an average localization error of only 12 ± 8 mm, significantly improving spatial localization accuracy. Then, He et al. (30) developed a novel algorithm based on these regression-based methods, combining multiple ML models, including SVM, random forests (RF), gradient-boosted decision trees (GBDT), and Gaussian Naive Bayes (GNB) to automatically extract and classify PVCs across 11 regions of the entire ventricle, reporting an overall diagnostic accuracy ranging from 70.7% to 74.1% (30). However, the ECG waveforms in the RVOT and right ventricular anterior wall regions are similar, resulting in suboptimal classification outcomes. Therefore, additional classification strategies are required to achieve targeted differentiation. Building upon the work of He et al., Wang et al. (31) proposed an innovative “two-step classification” ML method in 2024. They first classified PVCs into 11 categories, then applied a binary classifier specifically to the ambiguous subgroups. This method achieves precise localization of origin points throughout the entire ventricle through optimized feature extraction and a computationally efficient model.

These traditional ML approaches offer two key advantages, interpretability and computational efficiency. One can directly trace the contribution of individual features to the model's decision, and these models require minimal computational resources. However, their performance is fundamentally constrained by the quality of feature engineering. When confronted with atypical ECG morphology or rare origins, these models often struggle. For example, He et al. acknowledged that their model got confused between the RVOT and the right ventricular anterior wall due to highly similar waveform characteristics (30).

DL emerged to address these limitations by automating feature learning, particularly for cases with complex or subtle ECG patterns. Compared with traditional ML methods, DL (particularly deep neural networks) has demonstrated significant advantages in handling large datasets and complex pattern recognition due to its powerful automatic feature extraction capabilities. Yang et al. (32) pioneered the application of DL in the field of PVC origin localization. By analyzing simulated data using two 2D CNNs, they successfully distinguished ventricular ectopic beats originating from different cardiac segments and from epicardial vs. endocardial sources, and proposed an origin localization algorithm based on network outputs, offering a new approach to non-invasive localization of arrhythmias (32). Nakamura et al. (33) further optimized the classification strategy, using a CNN to classify PVC origin into four categories: right/left and outflow tract/non-outflow tract, with performance superior to that of SVM models (33). In 2023, the ECGNet model developed by Zhang et al. (34) achieved automatic classification of six origin categories, including RVOT, LVOT, papillary muscles, annulus, left ventricular apex, and the His-Purkinje system based on 12-lead ECG.

The primary strength of DL lies in its capacity to detect subtle patterns that may elude human experts. However, this capability is accompanied by inherent limitations. In practice, most DL architectures function as “black boxes” offering limited transparency into their decision-making processes, and they typically require large, well-annotated datasets to achieve robust performance.

Researchers have made recent attempts to balance performance and clinical utility. Some newer work tries to keep DL's high accuracy while improving transparency or efficiency. Zheng et al. (35) developed a high-precision system using Extremely Randomized Trees, refining PVC origin prediction from three broad regions to 21 anatomical sites (35). In their single-center study of 545 patients, the 21-site scheme achieved a localization accuracy of 98.24%. However, this performance was attained within a dataset characterized by marked class imbalance, particularly with limited representation of rare origins such as the Summit. The study employed a hierarchical classification logic comprising four schemes. The first scheme classifies origins into three broad territories, the epicardium of left ventricular summit, the right ventricle, and the left ventricle. The second method distinguishes between left/right outflow tracts and non-outflow tract regions. The third predicts 18 anatomical sites, and the fourth scheme distinguishes 21 potential origin sites. While the fourth scheme achieved an accuracy rate of 98.24%, the first three schemes exceeded 99% accuracy. The model's performance was reported to significantly exceed that of human experts, but this finding comes from a single center internal test set and awaits external validation. Nevertheless, its automated feature extraction and clinically aligned hierarchical localization logic establish a high-precision paradigm for AI-based preoperative localization.

More recently, Guo et al. (36) introduced residual structures and attention mechanisms based on ECGNet to propose the PVCsNet model. In their single center study of 310 patients, the model improved the overall localization accuracy to 94.49%. However, the performance was constrained by marked class imbalance, with limited representation of rare origins such as the Summit. The network starts with four 3 × 3 convolutional layers to extract basic features, followed by residual connections and attention blocks. The attention part uses a Squeeze-and-Excitation (SE) block (with MaxPool and a reduction ratio of 4) to enable the model to focus on the most discriminative ECG segments for each type while suppressing noise from irrelevant leads or time windows. Additionally, the residual connections facilitate smoother network training. These results demonstrate the clinical potential of AI in PVC origin localization (36).

However, the data were collected from a single center under idealized conditions. Whether these models generalize to patients with SHD, prior ablation scars, or multiple comorbidities remains unknown. Traditional ML provides a pragmatic solution for settings with limited resources and for broad anatomical categorization, whereas DL proves essential for pinpointing rare sites like papillary muscles or the annulus, despite its black-box nature. The true restriction is the scarcity of large, multi-center datasets that reflect real-world complexity. Without such data, the clinical generalizability of these models remains uncertain.

A detailed summary of the AI models discussed in this review, including cohort sizes, validation methods, and predicted origin sites, is provided in Table 2.

**Table 2:** Summary of AI models for PVC origin localization, including cohort size, validation methods and predicted origin sites.

Study	Patients	Input/feature	Method	Location	Performance
Zheng et al. (14)	420	192 × 8 matrix	XGBoost	LVOT vs. RVOT	ACC: 97.62%
					SEN: 96.97%
					SPEC: 100%
Nakasone et al. (15)	80	12-lead ECG raw waveforms	CNN + Grad-CAM	LVOT vs. RVOT	ACC: 89.4%
					SEN: 95.2%
Shimojo et al. (16)	104	ECG morphology	Decision Tree	LVOT vs. RVOT	ACC: 90.60%
					SEN: 94.70%
Kujime et al. (17)	334	12-lead ECG raw signals	DNN	RVOT, LVOT, Neutral	ACC: 91.6%
					F1: 94.3%
					AUC: 84.8%
					Neutral Rate: 28.4%
Chang et al. (18)	731	12 × 1,204 image	CNN	RVOT, LVOT, LV Summit	ACC: 98.91%
Yokokawa et al. (28)	34	Digitized 12-lead pacing QRS morphology	SVM	10 LV segments	ACC: 71.00%
Sapp et al. (29)	38	QRS integral	Template Matching + Multiple Linear Regression	16 LV Segments	LE: 12 ± 8 mm
He et al. (30)	249	QRS morphology	GBDT, GNB, RF, SVM	11 regions across whole ventricle	ACC SVM: 74.10%
					RF: 72.70%
					GBDT: 72.70%
					GNB: 70.70%
Wang et al. (31)	249	QRS morphology	GBDT, KNN, RF, SVM	11 regions (binary for overlapping)	ACC: 76.84%
Zheng et al. (32)	545	QRS complex	98 ML Methods	whole ventricle: 21 anatomical sites	ACC: 98.24%
Yang et al. (33)	9	16 × 16 matrix	CNN	LV, RV	LE: 11.00 mm
Nakamura et al. (34)	111	12 × 260 matrix	CNN, SVM	Right/Left, outflow/non-outflow	ACC SVM: 85.00%
					CNN: 80.00%
Zhang et al. (35)	310	12-lead ECG images	CNN (ECGNet)	6 categories (RVOT, LVOT, PM, LV apex, summit, HPS)	ACC: 91.74%
Guo et al. (36)	310	12-lead ECG images	CNN + residual + attention (PVCsNet)	6 categories (RVOT, LVOT, PM, VA, summit, HPS)	ACC: 94.49%

RVOT, right ventricular outflow Tract; LVOT, left ventricular outflow tract; LV, left ventricular; RV, right ventricular; PM, papillary muscle; VA, valvular annulus; HPS, his-purkinje system; Grad-CAM, gradient-weighted class activation mapping; XGBoost, eXtreme gradient boosting; DNN, deep neural network; GNB, gaussian naive bayes; SVM, support vector machine; CNN, convolutional neural network; GBDT, gradient boosting decision trees; RF, random forest; SEN, sensitivity; SPEC, specificity; ACC, accuracy; LE, localization error; F1, F1-score.

## 4 Prospects and challenges of AI for PVC origin localization

AI is advancing rapidly in the field of PVC origin localization, but there are also many challenges.

Data quality and standardization are critical issues. While large-scale, well-annotated PVC datasets are essential for AI models, existing collections suffer from inconsistent annotation protocols and lack of multicenter diversity (37). Variations in cardiac anatomy, ECG device configurations, and operational protocols collectively contribute to discrepancies between recorded ECG features and the actual origin site. Therefore, sole reliance on expert labeling is inadequate. Future research should establish standardized annotation protocols by adapting established cardiac segmentation models for PVC origin annotation, and mandate transparent reporting of recording equipment specifications and patient anatomy.

Beyond data limitations, algorithm robustness requires further refinement. Current AI algorithms lack robustness across the spectrum of PVC morphologies. Many models are trained on relatively homogeneous populations, leading to performance degradation when faced with atypical ECG patterns or SHD. Enhanced generalization capabilities are essential for handling the complex and diverse PVC patterns encountered in clinical practice (38).

Interpretability remains a major barrier. The black-box nature of current AI models severely limits their clinical acceptance. The decision-making logic and rationale behind an algorithm’s localization of a specific origin are often difficult to comprehend, which undermines clinicians’ confidence in the results and may consequently diminish the algorithm’s value as a reference in ablation decision-making. Merely advocating for further research is insufficient. Although interpretability methods like gradient-weighted class activation mapping (Grad-CAM) are available, they require rigorous clinical validation. Future model design should prioritize explainability from the initial development phase, rather than treating it as an afterthought (39).

Real-world validation and clinical workflow are largely missing. Most studies have only tested their models on internal data from a single center, often with just a few hundred patients and standard 12-lead ECGs. Validation using Holter data, larger cohorts, or multicenter external datasets is rare. Patients with SHD, such as ischemic cardiomyopathy or arrhythmogenic right ventricular cardiomyopathy (ARVC), have been excluded from most studies. AI models trained on idiopathic PVCs often perform poorly in SHD patients. This is primarily due to pathological ventricular scar generation, which leads to slowed conduction and anisotropy, resulting in fragmented or widened QRS complexes on the ECG. These pathological changes completely disrupt the vector patterns of normal ventricular depolarization, rendering models trained on structurally normal hearts unreliable when applied to SHD data.

For these complex yet common clinical scenarios, the evidence base is thin. While most of these models are designed for preoperative PVC origin localization, the literature lacks data on practical deployment aspects, such as how they would be integrated into real-world clinical workflows, what computational latency they would introduce, or how user-friendly they would be for clinicians. Without proper validation on diverse, real-world populations and a clear picture of the intended clinical workflow, high accuracy metrics may lack substantive relevance for actual clinical practice. Consequently, future investigations must prioritize large-scale, multicenter validation studies, the development of datasets reflecting real-world complexity, and a transparent reporting of implementation metrics such as computational latency and usability.

Awareness and education remain limited. Many scientists, clinicians, and members of the public have a limited understanding of AI principles and applications, which hinders the promotion and clinical translation (40). Targeted educational initiatives are crucial to increase understanding and engagement (5).

Beyond these general limitations, electrophysiology (EP)-specific hurdles present additional challenges for AI application in PVC localization. Currently, most AI models are trained using preoperative static ECGs. During catheter ablation, mechanical stimulation from the catheter or catheter ablation can induce dynamic shifts in the PVC exit sites, rendering preoperative predictions unreliable. Future AI systems should not only be capable of analyzing static ECG data but also integrate with 3D mapping systems, such as Carto or Ensite, to enable continuous real-time updates of predicted PVC origins. In addition, AI-assisted real-time pace-mapping can rapidly and objectively assess the similarity between 12-lead QRS morphology and target PVCs, generating quantitative similarity scores that surpass manual evaluation. The integration of AI with intraoperative mapping is essential to enhance localization accuracy, support clinical decision-making, and realize the practical clinical value of AI in electrophysiology.

## 5 Conclusion

This review traces the evolution of PVC localization from traditional ECG criteria to contemporary AI methodologies. A comparative analysis of these paradigms reveals complementary strengths and limitations across three key dimensions: accuracy, reproducibility and usability. In terms of accuracy, AI models have reported superior performance in single center controlled studies. However, this advantage lacks robust external validation. With respect to reproducibility, AI eliminates the inter-observer variability inherent in manual ECG analysis, but its performance has not been validated across multi-center studies or diverse clinical environments. Regarding usability, traditional criteria retain advantages in interpretability and immediate clinical integration, whereas the black-box nature of advanced AI models and their untested integration into clinical workflows limit their practical application in clinical settings.

In the future, the most promising path forward appears to be the integration of AI's pattern-recognition capabilities with the traditional, interpretable framework of clinical reasoning. Moreover, AI applications in cardiology should focus on facilitating efficient analysis and developing predictive models specifically tailored to PVC management, ultimately advancing the practice of precision medicine.

## Data Availability

The original contributions presented in the study are included in the article/Supplementary Material, further inquiries can be directed to the corresponding author.
